# Medical diaspora: an underused entity in low- and middle-income countries’ health system development

**DOI:** 10.1186/s12960-019-0393-1

**Published:** 2019-07-15

**Authors:** Seble Frehywot, Chulwoo Park, Alexandra Infanzon

**Affiliations:** 1 Milken Institute School of Public Health, The George Washington University, 950 New Hampshire Ave. NW, Washington DC, 20052 United States of America; 2 Lenoir-Rhyne University, 625 7th Ave NE, Hickory, NC 28601 United States of America

**Keywords:** Medical diaspora, Low- and middle-income countries, Capacity development

## Abstract

**Background:**

At present, over 215 million people live outside their countries of birth, many of which are referred to as diaspora—those that live in host countries but maintain strong sentimental and material links with their countries of origin, their homelands. The critical shortage of Human Resources for Health (HRH) in many developing countries remains a barrier to attaining their health system goals. Usage of medical diaspora can be one way to meet this need. A growing number of policy-makers have come to acknowledge that medical diaspora can play a vital role in the development of their homeland’s health workforce capacity. To date, no inventory of low- and middle-income countries (LMIC) medical diaspora organizations has been done. This paper intends to develop an inventory that is as complete as possible, of the names of the LMIC medical diaspora organizations in the United States of America, the United Kingdom, Canada, and Australia and addresses their interests and roles in building the health system of their country of origin.

**Methods:**

The researchers utilized six steps for their research methodology: (1) development of rationale for choosing the four destination countries (the United States of America, the United Kingdom, Canada, and Australia); (2) identification of low- and middle-income countries (LMIC); (3) web search for the name of LMIC medical diaspora organization in the United States of America, the United Kingdom, Canada, and Australia through the search engines of PubMed, Scopus, Google, Google Scholar, and LexisNexis; (4) development of inclusion and exclusion criteria and creation of a medical diaspora organizations’ inventory list (Table 1) and corresponding maps (Figures 1, 2, and 3). Using decision criteria, reviewers narrowed the number to a final 89 organizations; (5) synthesis of information to collect the general as well as the unique roles the medical diaspora organizations play in building health systems; and (6) developing inventory of respective LMIC governments’ diaspora offices (Table 2) to identify units/departments that facilitate diaspora’s work.

**Result:**

In total, the authors found 89 medical diaspora organizations in 4 main countries: in the United States of America 60, in the United Kingdom 24, in Australia 3, and in Canada 2. These medical diaspora organizations tend to have three focuses: providing healthcare services, training, and when needed humanitarian aid to their home country; creating a social or professional network of migrant physicians (i.e., simply to bring together people with an ethnic and professional commonality) and; supplying improved and culturally sensitive healthcare to the migrant population within the host country. Sixty-eight LMIC countries have established a diaspora office within their government office. It is also equally important to note that many policy-makers may lack knowledge of models for medical diaspora engagement or of valuable lessons learned by other governments about working with diaspora.

**Conclusions:**

The medical diaspora remains an underutilized resource in both health systems policy formulation and program implementation.

## Background

There are various definitions of the term diaspora, many of which have evolved over time. Once exclusively used in a context-bound way, describing that of Jewish history and the plight of Jewish people being dispersed “among the nations,” the folk term became generalized on a grand (global) scale in the late twentieth century. Since the 1970s, “diaspora” has been increasingly used to denote people living far away from their ancestral or former homeland, which is reflected in Sheffer’s definition of the modern diaspora as “ethnic minority groups of migrant origins residing and acting in host countries but maintaining strong sentimental and material links with their countries of origin, their homelands” [1]. For many developing countries, remittances from the diaspora are an important source of foreign exchange, surpassing earnings from major exports, and covering a substantial portion of imports [2]. The history of the semantics of the term “diaspora” points to several changes of the term’s meaning. As is fairly well known, “diaspora” is a Greek term. The noun dÝaw o € is a derivation from the Greek composite verb “dia-” and “speirein” (dÝaw e€Ý eÝn, infinitive), adopting meanings of “to scatter,” “to spread,” or “to disperse” [3]. For the purpose of this paper, medical diaspora is defined by the authors as physicians that have migrated from their country of origin to another country.

The Sustainable Development Goals (SDGs), which build upon the Millennium Development Goals (MDGs), emphasize a cross-thematic framework in the post-2015 development agenda [4]. Although health is not explicitly mentioned, the SDGs demonstrate the important linkages between health and development. To achieve these ambitious targets, the international community around the globe has committed to investing heavily in health systems to support these efforts, particularly in training and retention of health workers. However, the critical shortage of Human Resources for Health (HRH) in many developing countries remains a barrier to optimizing these efforts [5, 6]. Usage of medical diaspora can be one way to meet this need. Diaspora groups possess vital knowledge of the social and cultural context of their homelands. Over the past few years, the contributions of migrants and diaspora to sustainable development in their countries of origin and destination have been acknowledged by the 2030 Agenda for Sustainable Development, the New York Declaration for Refugees and Migrants and the Summits of the Global Forum on Migration and Development [7]. In addition, countries may want to advance the utilization of the medical diaspora as a way of countering the negative impacts created by the medical migration. Return migrants, in particular, bring back their skills and work experience from abroad boosting productivity [8].

To date, no inventory of low- and middle-income countries’ (LMIC) medical diaspora organizations, based on their location, specialty of work, and the prospect and feasibility of using them for the capacity development of the health workforce in LMIC has been done. The authors set out with the research question of “do medical diaspora organizations exist in these four developed countries and if they do, what are the key roles of these organizations?”

Based on this, this paper aims to develop an inventory that is as complete as possible, of the roles of the LMIC medical diaspora organizations in building the health care system of their country of origin.

## Methods

The method for the paper is comprised of six steps:

1. Development of rationale for choosing the four destination countries (the United States of America, the United Kingdom, Canada, and Australia)

From the outset, for two main reasons, the focus of this research has been on four anglophone destination countries: the United States of America, the United Kingdom, Canada, and Australia. This is because of two reasons. First, in many cases medical diaspora are actively recruited by the wealthy nations of Europe, North America, Australia, and elsewhere [9], and second specifically, because international medical graduates constitute between 23 and 28 percent of physicians in the United States of America, the United Kingdom, Canada, and Australia, and lower-income countries supply between 40 and 75% of these international medical graduates [10].

2. Identification of low- and middle-income countries

This list was obtained from the World Bank website [11].

3. Web search

For each LMIC country, search for the name of medical diaspora organization in the United States, the United Kingdom, Canada, and Australia was conducted through PubMed, Scopus, Google, Google Scholar, and LexisNexis. Each individual LMIC country was connected with the search terms of medical diaspora organization, medical diaspora engagement, medical diaspora services, medical diaspora initiatives, and medical diaspora contributions using Boolean Operation “AND” and “OR.” Through this, a list of 130 medical diaspora organizations was identified.

4. Development of inclusion and exclusion criteria and creation of a medical diaspora organizations’ inventory list and corresponding maps

By using inclusion and exclusion criteria, the list of 130 medical diaspora organizations was narrowed down to 89 medical diaspora organizations.

- Inclusion criteria:

Medical diaspora organizations that are located in the following four countries: Australia, Canada, the United Kingdom, and the United States of America.

Medical diaspora organizations’ members are from a specific LMIC country.

The organization mainly consists of medical doctors.

The organization name clearly includes word associated with the medical profession.

The medical diaspora organizations headquarter office has an active webpage and indicates the actual address of the headquarter office.

- Exclusion criteria:

Biomedical science, scientist, or engineer diaspora organizations

Nurse or pharmacist diaspora organizations

Organizations of regions or chapters: Many of organizations had branches across the country, such as in the name of regions or chapters, but it was worth including only the representative one, such as headquarter, to maintain the simplicity of the medical diaspora organization list (see Table 1).

- Exception:

**Table 1:** The list of medical diaspora organizations

Medical diaspora organizations (in alphabetical order)	URL site
1. Academy of Persian American Physicians	http://persianphysician.org
2. Afghan Medical Association of America	http://www.afghanmed.org
3. Afghan Medical Professionals Association of America	http://www.ampaa.org
4. Albanian American Medical Society, Inc.	https://www.albamedsociety.org
5. American Association of Cardiologists of Indian Origin	http://www.aacio.org
6. American Association of Physicians of Indian Origin	https://aapiusa.org
7. American Board Certified Doctors for Egypt	http://www.doctorsforegypt.com
8. American Lebanese Medical Association	https://www.almamater.org
9. American Nepal Medical Foundation	https://www.anmf.org
10. American Society of Indian Plastic Surgeons	https://www.facebook.com/American-Society-of-Indian-Plastic-Surgeons-189293321104812
11. American Ukrainian Medical Foundation	http://aumf.net
12. Argentine-American Medical Society	http://www.aams.us
13. Armenian American Medical Society	https://aamsc.org
14. Armenian Medical Society of Great Britain	http://www.accc.org.uk
15. Association of Afghan Healthcare Professionals UK	http://aahpuk.org
16. Association of Chinese American Physicians	http://www.acaponline.org
17. Association of Haitian Physicians Abroad	http://www.amhe.org/index.html
18. Association of Kerala Medical Graduates	http://www.akmg.org
19. Association of Nepali Physicians in America	http://anpa-usa.org
20. Association of Nigerian Physicians in the Americas	https://anpa.org
21. Association of Pakistani Physicians and Surgeons of the UK	http://appsuk.org
22. Association of Philippine Physicians in America*	Although this representative organization has no website, there are a number of its chapters in each state.
23. Association of Physicians of Pakistani Descent of North America	http://appna.org
24. Australia Myanmar Medical Association	http://www.au-mma.org
25. Bangladesh Medical Association of North America	https://www.bmana.org
26. Bangladesh Medical Association UK	http://www.bmsuk.org.uk
27. Bolivian American Medical Society	http://www.bolivianamericanmedicalsociety.com
28. British Association of Physicians of Indian Origin	https://www.bapio.co.uk
29. British Iranian Medical Association	https://www.facebook.com/BIMAUK
30. British Islamic Medical Association (for Sudanese descent)	http://www.britishima.org
31. Burmese American Medical Association	http://www.bamausa.org
32. Burmese Doctors and Dentists Association UK	https://www.facebook.com/BDDAUK/?ref=page_internal
33. Burmese Medical Association Australia	https://www.facebook.com/Burmese-Medical-Association-Australia-BMAA-10150140888940538
34. Burmese Medical Association of North America	http://bma-na.com
35. Cambodian Health Professionals Association of America	https://www.chpaa.org
36. Cameroon Doctors UK	https://www.camdocuk.org/home
37. Canadian Association of Nigerian Physicians and Dentists	http://canpad.org
38. Chinese American Medical Society	http://chineseamericanmedicalsociety.cloverpad.org
39. Chinese American Physicians Society	http://www.caps-ca.org
40. Dominican Medical Association	http://www.dmanewyork.com
41. Egyptian American Medical Association	http://www.e-ama.org
42. Egyptian American Medical Society	http://www.egyptianamericanms.com
43. Egyptian Association for American Medical Training and Research	www.eamtar.com
44. Egyptian Medical Society UK	http://www.egyptianmedical.org.uk
45. Ethiopian North American Health Professionals Association	http://enahpa.org
46. Ghana Physicians and Surgeons Foundation	https://www.ghanaphysicians.org
47. Ghanaian Doctors & Dentists Association	https://www.gddauk.org
48. Iranian American Medical Association	https://www.iama.org
49. Iranian Medical Society	http://www.iranianmedicalsociety.org/Index.cfm
50. Iranian Medical Society UK	http://www.iranianmedicalsociety.org.uk
51. Iranian-American Medical Society of Greater Washington (Iranian Medical & Dental Society of the Greater Wash-Balt Metro Area)	http://www.iamsgw.org
52. Iraqi Medical Association UK	https://www.imauk.com
53. Iraqi Medical Sciences Association USA	https://www.imsausa.org
54. Medical Associations of Nigerians Across Great Britain	http://www.mansag.org
55. Myanmar American Medical Education Society	http://mamesociety.org
56. Nepalese Doctors Association	http://ndauk.org.uk/index.php/home
57. Nicaraguan American Medical Association	http://www.nicamed.com
58. Nigeria American Medical Foundation International	https://namfi.org
59. North American Taiwanese Medical Association	https://www.natma.org
60. Northern Indian Medical & Dental Association of Canada	http://www.nimdac.com
61. Organization of Sierra Leonean Healthcare Professionals Abroad	http://www.toshpa.org.uk/index.htm
62. Pakistan Medical Association UK	https://www.facebook.com/pages/category/Medical-Company/Pakistan-Medical-Association-UK-346946281999626
63. People To People (Ethiopia)	https://p2pbridge.wordpress.com
64. Peruvian American Medical Society	http://www.pams.org/
65. Philippine Medical Association in America*	Although this representative organization has no website, there are a number of its chapters in each state.
66. Rajasthan Medical Alumni Association	http://www.rajmaai.com
67. Romanian Medical Association of America	https://www.facebook.com/Romanian-Medical-Association-of-America-250257948368293
68. Romanian Medical Society UK	https://www.facebook.com/romanianmedicalsociety
69. Russian American Medical Association	http://www.russiandoctors.org/en
70. Serbian American Medical and Dental Society	http://www.samds.org
71. Serbian American Medical Association	http://www.serbianama.org
72. Society of Philippine Surgeons in America	http://www.spsatoday.com
73. South Australian Sri Lankan Doctors Association	https://sasda.org.au
74. Sri Lanka Medical Association of North America*	Although this representative organization has no website, there are a number of its chapters in each state.
75. Sri Lankan Medical and Dental Association of the UK	https://www.srilankan-mda.org.uk
76. Sudanese American Medical Association	https://www.sama-sd.org
77. Sudanese Junior Doctors Association UK	https://www.sjda.uk
78. Sudanese Medical Association UK and Ireland	https://www.facebook.com/groups/sma.ukandire/?ref=group_header
79. Syrian American Medical Society Foundation	https://www.sams-usa.net
80. Syrian British Medical Society	http://sb-ms.org
81. Tanzania UK Healthcare Diaspora Association	http://tuheda.org
82. Thai Physicians Association of America	https://tpaa.us
83. Ukrainian Medical Association of North America	https://www.umana.org
84. Ukrainian Medical Association of the UK	http://www.umauk.com
85. United States Colombian Medical Association	https://uscma.wordpress.com
86. University of Santo Tomas Medical Alumni Association in America (Philippine)	https://ustmaaamerica.wildapricot.org
87. Venezuelan American Medical Association	https://www.vamainfo.com
88. Vietnamese American Medical Association	http://www.vamausa.org
89. Vietnamese Physicians Association*	Although this representative organization has no website, there are a number of its chapters in each state.

There are four medical diaspora organizations that have been included because they did not satisfy all inclusion criteria: Association of Philippine Physicians in America, Philippine Medical Association in America, Sri Lanka Medical Association of North America, and Vietnamese Physicians Association. Although the headquarters of those organizations had no website, there were a number of their chapters with viable websites across the United States of America.

**Fig. 1:**
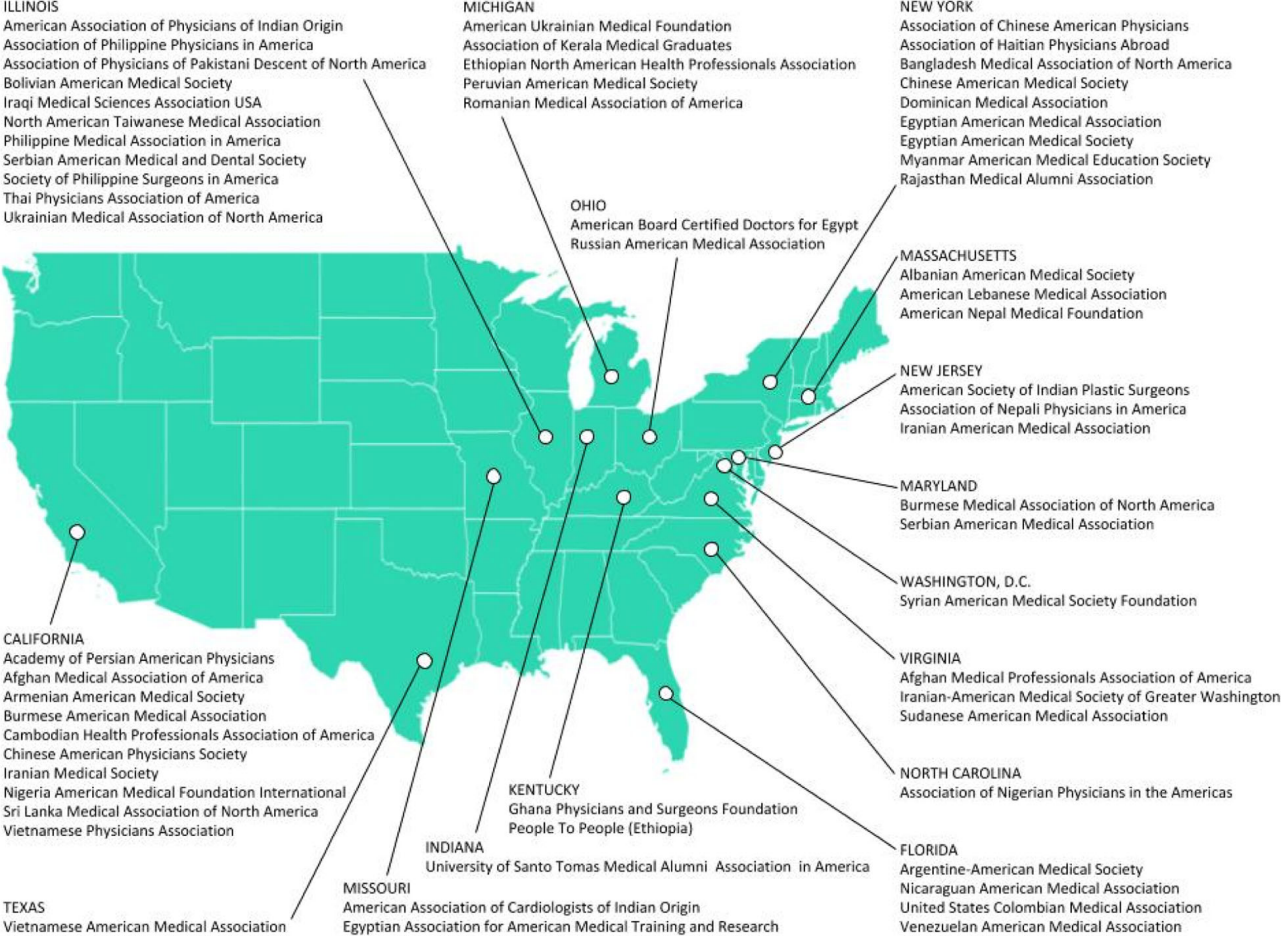
Medical diaspora organizations in the United States of America

**Fig. 2:**
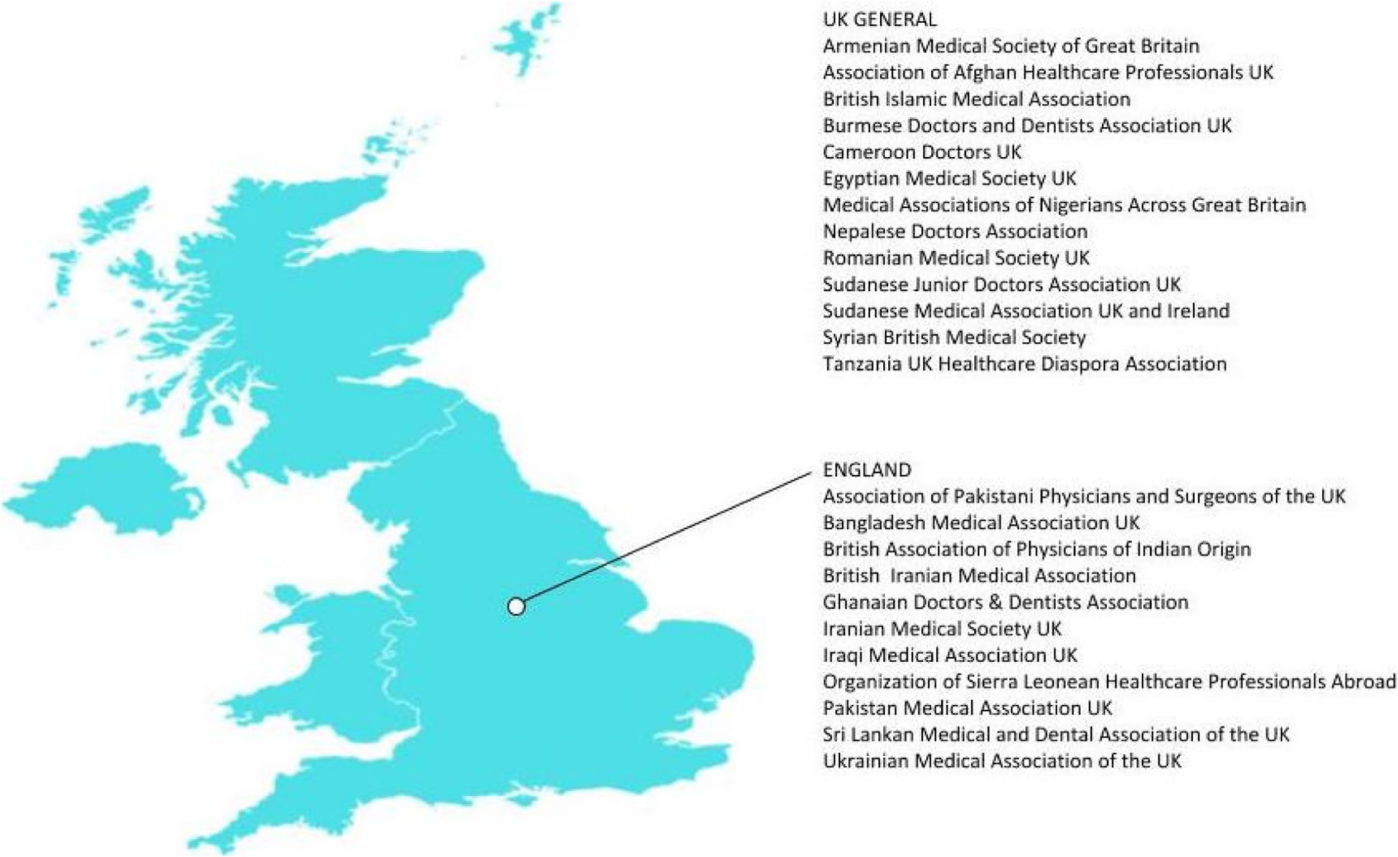
Medical diaspora organizations in the United Kingdom

**Fig. 3:**
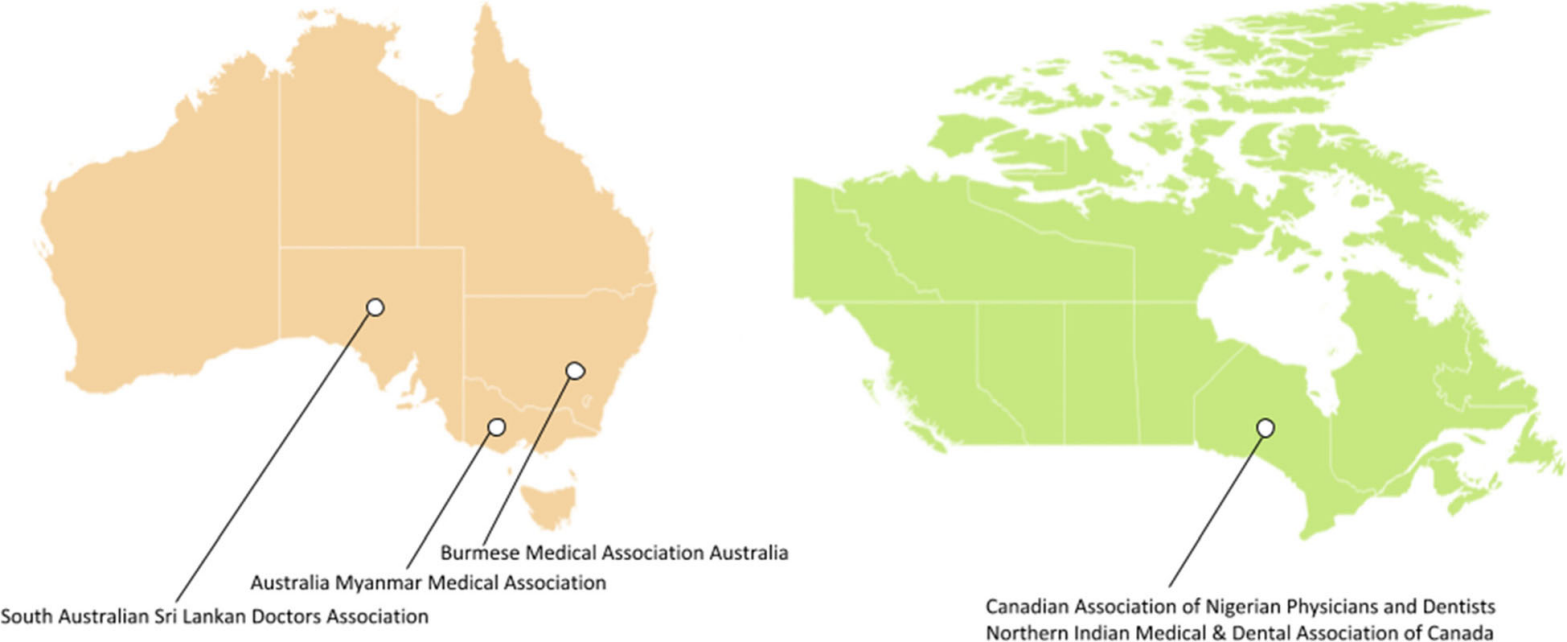
Medical diaspora organizations in Australia and Canada

Once the list was finalized, an inventory list of the medical diaspora organizations in the United States of America, the United Kingdom, Canada, and Australia was developed together with a corresponding map (see Table 1 and Figs. 1, 2, and 3).

5. Synthesis of information

Based on the research question, each medical diaspora website and the articles we found about it was searched to collect the work that the particular institution engages in and the general as well as the unique roles the medical diaspora organization plays in building health systems.

6. Developing inventory of respective LMIC governments’ diaspora offices

For each LMIC country, a search of the respective LMIC Ministry of Foreign Affairs sites (or its equivalent) was conducted to identify the existence, contact information and details of government units or departments that facilitate diaspora’s work. A per LMIC country inventory list of this units was created (see Table 2).

**Table 2:** Table of country diaspora offices within the respective Ministry of Foreign Affairs

Country	Engagement office	Website	Year established
Afghanistan			Not available
Albania	Ministry of Foreign Affairs, Diaspora Department		Not available
Algeria	Ministry of National Solidarity, Family and the National Community Abroad		Not available
American Samoa			Not available
Angola	Ministry of Foreign Affairs Institute of the Angolan Communities Abroad and Consulate Affairs	http://www.mirex.gov.ao	Not available
Armenia	Ministry of Diaspora		Not available
Azerbaijan	State Committee on Affairs of the Diaspora	http://www.azerbaijans.com/content_556_en.html	2002
Bangladesh	Ministry of Expatriates’ Welfare and Overseas Employment (MEWOE)	http://www.probashi.gov.bd	2001
Belarus			
Belize			
Benin	Ministry for Foreign Affairs, African Integration, the Francophone Community, and Beninese Abroad	Not available	Not available
Bhutan			Not available
Bolivia (Plurinational State of)			Not available
Bosnia Herzegovina	Ministry of Human Rights and Refugees: Department of Diaspora	http://www.mhrr.gov.ba/iseljenistvo/aktuelnosti/Archive.aspx?langTag=bs-BA&template_id=128&pageIndex=1	Not available
Botswana			Not available
Brazil	Ministry of Foreign Affairs, Undersecretary General for Brazilian Communities Abroad	http://www.brasileirosnomundo.itamaraty.gov.br	Not available
Bulgaria	State Agency for Bulgarians Abroad	http://www.aba.government.bg/?show=10	2000
Burkina Faso	Ministere Des Affaires Etrangres Et De La Cooperation Regionale	http://www.mae.gov.bf	2007
Burundi	Ministry of Foreign Affairs, Directorate of Diaspora	Not available	Not available
Cambodia		Not available	Not available
Cameroon	Not available	Not available	Not available
Cape Verde	Not available	Not available	Not available
Central African Republic	Not available	Not available	Not available
Chad	Not available	Not available	Not available
China	State Council, Overseas Chinese Affairs Office of the State Council; Overseas Chinese Affairs Committee The Overseas Chinese Affairs Office (SOCAO) of Shanghai Municipal People’s Government	http://www.overseas.sh.cn	1978, renamed 1980
Colombia	Colombia Nos Une, a program of the Bureau of Consular Affairs and Colombian Communities Abroad in the Ministry of Foreign Affairs	http://www.redescolombia.org	Not available
Comoros	Ministry of External Relations and Cooperation of the Diaspora		Not available
Congo, Dem. Rep. of			Not available
Congo, Rep. of			Not available
Costa Rica			Not available
Cote D’Ivoire			Not available
Cuba			Not available
Djibouti			Not available
Dominica	Ministry of Trade, Industry, Consumer, Diaspora Affairs	http://www.dominica.gov.dm/employment-trade-industry-and-diaspora-affairs	Not available
Dominican Republic	National Presidential Council for Dominican Communities Abroad		Not available
Ecuador			Not available
Egypt	Ministry of Manpower and Emigration, Emigration Sector Higher Committee on Migration		Not available
El Salvador	Ministry of Foreign Affairs, Vice Ministry for Salvadorans Abroad National Secretariat for Migrants (various states)	http://www.rree.gob.sv	2004
Eritrea	Ministry of Foreign Affairs, Department of Eritreans Abroad	http://www.mfa.gov.et/Diaspora/more.php?newsid=7	Late 1990’s
Ethiopia	Ministry of Foreign Affairs, Diaspora Affairs Directorate General; Ministry of Capacity Building, Diaspora Coordinating Office	http://www.mfa.gov.et/Diaspora/more.php?newsid=7	2002
Fiji			Not available
Gabon			Not available
The Gambia	Ministry of Foreign Affairs, International Cooperation & Gambians Abroad	http://www.mofa.gov.gm/index.php?option=com_content&view=article&id=58&Itemid=83	Not available
Georgia	State Ministry for Diaspora Issues	http://diaspora.gov.ge/index.php?lang_id=ENG	2008
Ghana	Ministry of Interior, National Migration Unit	http://www.mfa.gov.gh/index.php?id=1	2003
Grenada	Office of Diaspora Affairs, division of Ministry of Foreign Affairs	http://www.oxonwebmaster.com/mfa/index.php?q=diaspora	2009
Guatemala	National Council for Migrants from Guatemala		Not available
Guinea	Ministry of Foreign Affairs and Guineans Abroad	No website but phone number was found: Phone +224 30 45 12 70	Not available
Guinea-Bissau			Not available
Guyana	Ministry of Foreign Affairs Diaspora Unit	http://www.minfor.gov.gy/index.php?option=com_content&task=view&id=70&Itemid=120	Not available
Haiti	Ministry of Haitians Living Abroad	http://mhave.gouv.ht	1995
Honduras			
India	Ministry of Overseas Indian Affairs Government of Kerala, Department of Non-Resident Keralites’ Affairs; Government of Gujarat, Non-Resident Indian Division	http://moia.gov.in	2004
Indonesia	Ministry of Manpower and Transmigration	http://indonesia.go.id/en/ministries/ministers/ministry-of-manpower-and-transmigration/1644-profile/194-kementeriann-tenaga-kerja-dan-transmigrasi.html	1983
Iran			
Iraq	Ministry of Migration and Displaced		
Jamaica	Diaspora and Consular Affairs Office	http://www.mfaft.gov.jm/jm/about-us/departments-units/diaspora-consular-affairs	1993
Jordan	Department of Consular Affairs and Expatriates	http://www.mfa.gov.jo/ar/%D8%A7%D9%84%D9%82%D8%A7%D8%A6%D9%85%D8%A9%D8%A7%D9%84%D8%B1%D8%A6%D9%8A%D8%B3%D9%8A%D8%A9/%D8%A7%D9%84%D8%B5%D9%81%D8%AD%D8%A9%D8%A7%D9%84%D8%B1%D8%A6%D9%8A%D8%B3%D9%8A%D8%A9/tabid/71/Default.aspx	Not available
Kazakhstan			
Kenya	Ministry of Foreign Affairs: Diaspora Affairs Directorate i.e. International Jobs and Diaspora Office (Directorate)	http://www.kenyaembassytlv.org.il/209618/MINISTRY-OF-FOREIGN-AFFAIRS-ON-DIASPORA	2007
Kiribati			Not available
Korea, Dem. Rep. of			Not available
Kosovo	“Services for Citizens” Ministry of Foreign Affairs	http://www.mfa-ks.net/?page=2,151	Not available
Kyrgyz Republic			Not available
Lao PDR			Not available
Lebanon	Ministry of Foreign Affairs and Emigrants		Not available
Lesotho	Association: Diaspora Alliance	http://lesothopretoria.com/?trade_14	Not available
Liberia	Ministry of states with no portfolio	http://www.emansion.gov.lr/2content.php?sub=32&related=21&third=32&pg=sp http://www.mofa.gov.lr/public2/doc/EOI%20FOR%20DIASPORA%20ADVISOR(Bank%20Comments)%20(1).pdf	1972
Libya			Not available
Macedonia, FYR	Agency for Emigration		Not available
Madagascar	Ministère des Affaires Etrangères- “Diaspora and Economic Development”	http://mae.gov.mg/#	Not available
Malawi	Ministry of Foreign Affairs—Diaspora Services	http://www.foreignaffairs.gov.mw/index.php/diaspora-services/overview	Not available
Malaysia, Marshall Islands			Not available
Maldives			Not available
Mali	Ministry of Malians Abroad and African Integration Consultation Framework on Migration High Council of Malians Abroad	http://www.ime.gob.mx	2002
Mauritania			Not available
Mauritius			Not available
Mexico	Secretariat of Foreign Affairs, Sub-secretariat for North America; Institute for Mexicans Abroad National Council on Mexican Communities Abroad National Coordination for State-level Migrant Affairs Offices (various states) Consultative Council of the Institute for Mexicans Abroad	http://www.ime.gob.mx	2002
Micronesia, Federated States of			Not available
Moldova	Bureau of Inter-Ethnic Relations has a Diaspora branch	http://www.bri.gov.md/index.php?pag=sec&id=122&l=en http://diaspora.md	2010
Mongolia			Not available
Montenegro			Not available
Morocco	Ministry Charged with the Moroccan Community Residing Abroad Interdepartmental Committees Hassan II Foundation for Moroccans Resident Abroad Council on the Moroccan Community Abroad		Not available
Mozambique	Institute for Mozambican Communities Living Abroad (INACE) -a department in the Ministry of Foreign Affairs	http://www.iom.int/cms/en/sites/iom/home/news-and-views/press-briefing-notes/pbn-2014b/pbn-listing/mozambique-launches-diaspora-eng.html	2014
Myanmar			Not available
Namibia			Not available
Nepal			Not available
Nicaragua			Not available
Niger	Ministry of African Integration and Nigerians Abroad Committee in Charge of Migration	http://www.messrs.ne/index.php?option=com_content&view=article&id=70:ministere-des-affaires-etrangeres-de-lintegration-africaine-et-des-nigeriens-a-lexterieur&catid=1:liste-des-appeles-du-service-civique-national-pro&Itemid=67	Not available
Nigeria	Technical Working Group and Interministerial Committee on Migration	http://www.nigeriadiasporaday.com/index.php/about/nnvs	2002
Pakistan	Ministry of Overseas Pakistanis	http://www.ophrd.gov.pk/gop/index.php?q=aHR0cDovLzE5Mi4xNjguNzAuMTM2L2hyZC9kZWZhdWx0LmFzcHg%3D	Not available
Papua New Guinea			Not available
Paraguay			Not available
Peru	Ministry of Foreign Affairs, Undersecretary for Peruvians Abroad Advisory Council	http://www.rree.gob.pe	2003
Philippines	Philippines Department of Labor, Overseas Workers Welfare Administration; Department of Labor, Philippine Overseas Employment Administration; Department of Foreign Affairs, Office of the Undersecretary for Migrant Workers’ AffairsOffice of the President, Commission on Filipinos Overseas; Committee on Overseas Workers Affairs	http://www.owwa.gov.ph https://cfo.gov.ph/	1981
Romania	Ministry of Foreign Affairs, Department for Relations with the Romanians Abroad	http://www.dprp.gov.ro/about-us	Not available
Russian Federation			Not available
Rwanda	Department on diaspora engagement within the Ministry of External Relations and Internal Cooperation (MINAFFET), Diaspora General Directorate (DGD) Rwandan Community Abroad (RCA)	http://www.minaffet.gov.rw/index.php?id=890	2008
Samoa			Not available
Sao Tome and Principe			Not available
Senegal	Ministry of Senegalese Abroad	http://www.diplomatie.gouv.sn	2003
Serbia	Ministry of Religion and Diaspora	http://www.mfa.gov.rs/en/consular-affairs/diaspora/diaspora-general-information	
Sierra Leone	Office of the President, Office of the Diaspora	http://www.diasporaaffairs.gov.sl/index.php?option=com_content&view=article&id=1&Itemid=2	2007
Solomon Islands			Not available
Somalia	Ministry for Diaspora and Community Affairs Office for Development and Partnership with the Puntland Diaspora Community	http://www.mfa.somaligov.net https://www.facebook.com/OfficeforDiasporaAffairs	2013
South Africa	Department of International Relations and Cooperation	http://www.dfa.gov.za	Not available
South Sudan	Diaspora Engagement Steering Committee for South Sudan	https://www.facebook.com/southsudandiasporaforhealth	2013
Sri Lanka	Ministry of Foreign Employment Promotion and Welfare	http://www.foreignemploymin.gov.lk	2010
St. Lucia			Not available
St. Vincent and the Grenadines			Not available
Sudan			Not available
Suriname			Not available
Swaziland			Not available
Syrian Arab Republic	Ministry of Foreign Affairs and Expatriates	http://www.moex.gov.sy	2011
Tajikistan			Not available
Tanzania	Minister for Foreign Affairs and International Cooperation: task force of stakeholders, which involves members from his ministry, Ministry of Labour and Employment, Planning Commission, Public Service Management and National Statistics Bureau, which will be in charge of monitoring and coordinating engagement Diaspora	http://www.foreign.go.tz	Not available
Thailand			Not available
Timor-Leste			Not available
Togo	No apparent dept, but entire section of website dedicated to Togolese diaspora affairs	http://www.republicoftogo.com/Toutes-les-rubriques/Diaspora	Not available
Tonga			Not available
Tunisia	Ministry of Social Affairs, Solidarity, and Tunisians Abroad		Not available
Turkey			Not available
Turkmenistan			Not available
Tuvalu			Not available
Uganda	Diaspora office in the ministry of foreign affairs	http://www.mofa.go.ug/data/smenu/16/Overview-and-Mandate.html	2007
Ukraine			Not available
Uzbekistan			Not available
Vanuatu			Not available
Venezuela, Bolivarian Republic of			Not available
Vietnam	The Committee on overseas Vietnamese affairs, under the Ministry of Foreign Affairs	http://www.mofa.gov.vn/en	2004
West Bank and Gaza			Not available
Yemen			Not available
Zambia	Office within Ministry of foreign affairs**	http://www.foreignaffairs.gov.zm/index.php	Not available
Zimbabwe			Not available
	Total: 68		

## Results

In total, we found 89 medical diaspora organizations: 60 in the United States of America (US), 24 in the United Kingdom (UK), 3 in Australia, and 2 in Canada. Most of them are based in the United States of America., followed by the United Kingdom, Australia, and Canada, while the others work on an international level [12] (see Table 1 and Figs. 1, 2, and 3). Moreover, a total of 41 LMIC countries of origin was found among which those that originated from Asia were 21, from Africa 8, from the Americas 8, and from Europe 4 (Table 1):

- Asia (21): Afghanistan, Armenia, Bangladesh, Burma, Cambodia, China, India, Iran, Iraq, Israel, Lebanon, Myanmar, Nepal, Pakistan, Philippines, Russia, Sri Lanka, Syria, Taiwan, Thailand, and Vietnam
- Africa (8): Cameroon, Egypt, Ethiopia, Ghana, Nigeria, Sierra Leone, Sudan, and Tanzania
- America (8): Argentina, Bolivia, Colombia, Dominican Republic, Haiti, Nicaragua, Peru, and Venezuela
- Europe (4): Albania, Romania, Serbia, and Ukraine

## Common attributes

### Improving medical resources in their home country

The common attribute shared by all medical diaspora organizations is the desire to improve medical resources in their home country, including building medical facilities in their home country. Almost all migrant health workers have professional ties with their countries of origin supporting health, education, and social structures, felt indebted to their countries of origin, felt obliged to help as they were once granted scholarships or training opportunities, and thus, wanted to improve life in their countries of origin [13]. The level of contribution of each medical diaspora organization or individual varies. For instance, while Abdalla et al. (2016) reported that the effectiveness of the Sudanese medial diaspora was “small magnitude, infrequent and not well organized [14],” Nwadiuko et al. (2016) concluded that U.S.-based Nigerian physicians’ strong belief in effectiveness of Nigerian medical agencies would contribute to medical service trips to Nigeria [15]. In another instance, Wojczewski et al. (2015) have shown that African medical doctors who left their home countries as refugees cannot engage in any form of return initiatives, either short or long-term [13].

#### African countries

The Nigerian American Medical Foundation International (NAMFI) aims to provide world-class tertiary medical care on Nigerian soil through a gradual three-phase program of a sustainable 20-year development plan [16]. The Sudanese Medical Association UK and Ireland (SMA) aims to provide advice and support to the colleagues responsible for health services, medical and health education in Sudan, and contribute to the transfer of modern technology, expertise, and scientific updates to Sudan through cooperation with professional bodies and health authorities in the country [17]. The diaspora organizations collaborate with health professionals in their home country to provide a pathway for the exchange of information between the countries. The Ethiopian North American Health Professionals Association achieves this by using distance learning, providing medical training, and sponsoring international medical fellowships for Ethiopian health providers [18]. Similarly, the Ghana Physicians and Surgeons Foundation of North America (GPSF) aims to provide quality training for healthcare professionals in Ghana by providing subscriptions to world-class medical journals, superb online educational tools, and teaching materials to medical colleges in Ghana [19].

#### Asian countries

The American Association of Physicians of Indian Origin (AAPI) has set up multiple AAPI clinics in India, which offer free and compassionate health care [20]. The Afghan Medical Professionals Association of America (AMPAA) aims at promoting medical education and research, providing educational assistance by means of teaching materials, training opportunities, and collaboration with Afghan medical professionals, and providing assistance in improving the quality of medical education in Afghanistan [21].

#### European countries

The Albanian American Medical Society aims to create and maintain a fostering educational environment between the public academic institutions in the Albanian populated territories and those in the United States of America wherein its members may meet to exchange medical knowledge and participate in continuing medical education [22]. The mission of the Armenian American Medical Society is to cultivate and develop professional, social, and friendly relations among its members, and to contribute towards the improvement of the health services rendered to the Armenian community in the Diaspora and Armenia [23]. The Ukrainian Medical Association of the United Kingdom aims at developing ties with academic and professional healthcare organizations in the United Kingdom and Ukraine to promote social, cultural, educational, and research activities [24]. The Romanian Medical Association of America encourages fostering the establishment of professional interactions between North American and Romanian physicians and scientists, medical societies, universities, and institutions [25].

### Building medical human resource capacity in their home country

Many LMIC countries have been plagued for years with brain drain. Shortages of medical school faculty are rampant in many sub-Saharan African countries [26]. Medical diaspora organizations try to address this in one form or the other.

#### African countries

The People to People works to mobilize the global Ethiopian medical diaspora to play an active role in mitigating the impact of brain drain [27]. One of the goals of The Medical Association of Nigerians Across Great Britain is to support the improvement and effectiveness of Nigeria’s health programs by developing and increasing the capacity of health professionals in Nigeria and overseas [28]. The Association of Nigerian Physicians in the Americas encourages the development of practical solutions to Nigerian health care problems through strategic initiatives and field activities within Nigeria [29].

#### Asian countries

The Nepalese Doctors’ Association in the United Kingdom aims to contribute to the development of health services in Nepal by establishing greater understanding and co-operation among the Nepalese doctors in the United Kingdom [30]. The America Nepal Medical Foundation supports the Nepali people’s ongoing efforts to enhance their health status and focuses on improving the quality of medical care, medical education, and medical research in Nepal [31]. The Afghan Medical Professionals Association of America aims at improving the current health status of the Afghan nation and extending medical and educational aid to Afghanistan [21]. The Bangladesh Medical Association of North America supports local immunization clinics and free clinics in Bangladesh [32]. The Syrian American Medical Society Foundation, in collaboration with local physicians in Syria, has launched training campaigns for doctors in Syria [33].

### New trend—web-based medical diaspora organization

While most medical diaspora organizations have their own websites that provide an in-depth and informative introduction about them, some organizations only have Facebook pages and do not maintain their websites anymore. For example, the Sudanese Medical Association UK and Ireland, The Romanian Medical Association of America, and The Burmese Doctors and Dentists Association UK, British Iranian Medical Association are still active through Facebook pages [17, 25, 34, 35]. This new trend is utilized to effectively gather young medical doctors, scholars, and researchers to share the information with each other in the era of internet networking.

### Diaspora engagement: government level

Governments around the world have been supporting diaspora institutions and create migration policy [36, 37]. As can be seen in Table 2, diaspora institutions are in the form of ministry, agency, department, council, bureau, or institute. A growing number of policy-makers have come to acknowledge that diasporas can play a vital role in the development of their homelands and some developing countries have established institutions within their countries to further facilitate ties with their diasporas systematically (see Table 2). Sixty-eight LMIC countries have established a diaspora office within their government office.

Many LMICs are developing strategies to harness the resources of diaspora groups to drive development. China and India have created an increasingly extensive diasporic infrastructure, combined with policies designed to attract investments, as well as emotionally bind the diaspora to their country of origin. For instance, the Government of India is providing incentives to its diaspora, such as the recognition of persons of Indian origin through a special ministry and arranging special conferences, and the organization of a research scientist scheme that encourages diaspora scientists to teach at Indian universities [38]. In China, diaspora engagement policy has involved aggressively recruiting the return of its highly skilled diaspora through a variety of employment and scholarship programs [39]. The approaches adopted by these Asian nations point to country-driven initiatives that are built on shared development objectives between the government and the diaspora, and underlined by comprehensive policies, administrative structures, and incentives to foster an enabling environment for mobilizing diaspora resources (expertise, investments, entrepreneurship, and corporate affiliations) around critical growth pillars [40].

Furthermore, more than 30 African countries have established diaspora-oriented institutions and ministries, and many have created permanent diaspora government offices [41] (Table 2).

These offices are often a part of their respective Ministries of Foreign Affairs, either at the ministry or the sub-ministry level. Many countries also utilize consular networks that serve as the primary mechanism to engage with diaspora.

The government of Ethiopia has established some legal status for the Ethiopian diaspora. Ethiopian diasporas who hold non-Ethiopian nationalities have been entitled to an “Ethiopian Origin ID Card” that grants them some of the rights and privileges of an Ethiopian national [42]. In 2007, the Government of Kenya established the Diaspora Affairs Directorate through the Ministry of Foreign Affairs. This office creates opportunities to mobilize diaspora to participate in the formulation of an overall diaspora engagement policy. As an example, the Government of Sierra Leone established the Directorate of Diaspora Affairs (Directorate) in the Ministry of Presidential and Public Affairs and the government has been explicit about its intention to utilize the diaspora network to fill gaps in the availability of medical doctors and nurses [43]. On the contrary, while many governments acknowledge the importance of diaspora engagement in development, many still lack the capacity to design effective policies and implement them on a meaningful scale [44].

## Discussion

Throughout the collection of medical diaspora organizations from LMICs, some trends were noticed. Medical diaspora organizations tend to have three focuses: providing healthcare services, training, and when needed humanitarian aid to their home country; creating a social or professional network of migrant physicians (i.e., simply to bring together people with an ethnic and professional commonality); and supplying improved and culturally sensitive healthcare to the migrant population within the host country. Ethnic groups that did not necessarily have a large or well-established population within the host country came from countries where serious health problems still exist and were the most likely to have the first focus. Most of the diaspora groups from African countries fell into this category. Some groups from South/Southeast Asian countries like Bangladesh, as well as groups from select Middle Eastern and Central/Eastern European countries, also had this focus. Diaspora groups with a strong emphasis on the second focus were mainly intended to help new migrants feel welcome, make friends and successfully find jobs in the new country, as well as provide a forum for discussion of culturally relevant. In addition, these diaspora organizations are mainly used as a networking source to facilitate the exchange of information in the community, as opposed to providing services to their home countries [45]. Associations like these existed across diaspora groups from many continents, and mainly indicated a more casual, less explicitly purposed level of organization/mission. Ethnic groups that had a large and long-standing population within the host country were most likely to have the third focus—that of providing improved care to the migrant population. Such medical diaspora groups are exemplified by the Chinese, Thai, Vietnamese and Hispanic Americans, as well as Pakistanis and Indians in the United Kingdom.

In addition, there were other particular findings. Discovering which countries did not have medical diaspora groups was also sometimes interesting and may be instructive in identifying factors that contribute to an engaged diaspora. For example, though there are large migrant populations of South Africans across the world, no South African medical diaspora has been found. The same was true for Botswana and Namibia, although many other African countries, some with possibly with smaller migrant populations, had diaspora organizations. Crush et al. research on South African physicians in Canada shows that even though the South African medical diaspora in Canada who are mainly white, continue to assert their South African identity, they constitute a disengaged diaspora who are dissatisfied with the post-apartheid South African state [46]. Likewise, Chikanda and Dodson also showed that dissatisfaction with the political environment in the country of origin can have an adverse impact on the medical doctors’ desire to engage positively with the country of origin [47]. Other countries without medical diaspora organizations included most of the Pacific Islands, Indonesia, Lao, Libya, Tunisia, Senegal, and others. Why these communities do not have diaspora organizations while so many others similar or smaller countries do, might be an informative and interesting question to ask. In the same manner, it was interesting to note that only a handful of organizations have produced peer-reviewed articles. Instead, most of the work they perform is distributed among the community as newsletters. According to the research, the most notable work done in the field of publication is by the Syrian American Society, which sponsors a peer-reviewed online medical journal [48] It is also equally important to note that many policy-makers may not have comprehensive knowledge of their own diaspora’s development efforts and interests. Most, also, lack knowledge of models for diaspora engagement or of valuable lessons learned by other governments about working with diaspora. Thus, the health field related diaspora remains an underutilized resource and poorly-accounted-for factor in both health systems policy formulation and program implementation.

## Limitation

The following limitations in our research analysis made a potential impact on our research findings. First, there was a lack of available data on diaspora organizations, which limited the scope of our analysis and our ability to recognize additional common characteristics between the diaspora organizations. We have tried to do a very thorough search to find medical diaspora organizations, but there could be organizations that we have not found. Second, there is no previous global-level research study published on this matter. Without a proper foundation on this topic, we created the first analysis of its kind. To overcome these limitations, new researches are needed on medical and other health-related diaspora associations and their link to building various components of the health systems in LMIC. Third, we focused only on Anglophone recipient countries and did not include Francophone, Lusophone, Arabic speaking or other destination countries. Fourth, we acknowledge that there may be the possible existence of some medical diaspora organizations without an online presence. Finally, the authors believe more in-depth research is warranted to develop metrics to measure the effectiveness of medical diaspora organizations.

## Conclusions

Newland and Patrick identified the role of diasporas’ as supporting groups who pursue charitable enterprises and that their contribution has expanded beyond just investment inflows and remittances [49]. Diasporas are now viewed as important agents of change by countries of origin, donor agencies, and the international community. The skills of the diasporas can be tapped by establishing knowledge exchange networks. Some initiatives include mentor-sponsor programs, joint research projects, peer reviewer mechanisms, virtual return (through distance teaching and e-learning), and short-term visits and assignments. To increase the benefits of these activities, countries will have to map the gaps that they require to be filled in their health systems, survey the human resources available in their diasporas, create active networks, and develop specific activities and programs.

Despite this progress, work must continue to build the evidence base to develop the knowledge and capacity of engaging diaspora effectively at scale in a systematic and sustainable way. Countries and development partners should emphasize evaluation of efforts and mechanisms to share “what works” on a global platform to contribute to the global dialog. As we move forward, it will be increasingly important to utilize this evidence to build strategic partnerships between states, international organizations, civil society and private sector to create a framework for medical diaspora engagement and facilitate a transfer of resources and knowledge sharing.

## Data Availability

The data is available from the corresponding author.

## Abbreviations

DNA: Diaspora Networks Alliance
HRH: Human Resources for Health
IDEA: International Diaspora Engagement Alliance
LMIC: Low- and middle-income countries
MDGs: Millennium Development Goals
SDG: Sustainable Development Goals
WHO: World Health Organization
